# Roles and Interplay of SARS-CoV-2 Serology With Clinical Stages of Disease in COVID-19

**DOI:** 10.7759/cureus.15953

**Published:** 2021-06-27

**Authors:** Monica Mutyala, Ruhma Ali, Kok Hoe Chan, Aditya Patel, Chrystina Kiwan, Zareh Ekmekjian, Kalyan Koneru, Dhinesh V Reddy, Richard Miller, Maria Szabela, Jihad Slim

**Affiliations:** 1 Infectious Disease, Saint Michael's Medical Center, Newark, USA; 2 Internal Medicine, Saint Michael's Medical Center, Newark, USA; 3 Pulmonary and Critical Care, Saint Michael's Medical Center, Newark, USA; 4 Infectious Diseases, Saint Michael's Medical Center, Newark, USA

**Keywords:** coronavirus disease 2019, covid-19, serology, antibodies, sars-cov-2

## Abstract

Background

Currently, severe acute respiratory syndrome coronavirus 2 (SARS-CoV-2) serology is recommended only for seroprevalence. We think it could be useful in differentiating coronavirus disease 2019 (COVID-19) stages, which could in terms of helping improve our therapeutic interventions.

Methods

The medical records of adult patients admitted to the hospital with probable COVID-19 were extracted and analyzed. We excluded patients with no serology and no clear outcome at the end of data collection. Patient demographics, medical history, and biochemical and clinical data were retrieved.

Results

A total of 202 patients were included; 57% were males, the majority were Hispanic (45%), followed by African Americans (22%). Hypertension is the most common comorbidity, followed by diabetes mellitus and chronic kidney disease. We classified them into three groups based on their serology: subacute stage (47 patients) with both immunoglobulin M (IgM) and IgG negative; acute stage (116 patients) with IgM positive and late-stage (39 patients) with IgM negative and IgG positive. We found that elevated lactate dehydrogenase (LDH) and ferritin were present in the IgM+ and IgM-/IgG+ subgroups (p-value of 0.0061 and p-value 0.0013, respectively) while C-reactive protein (CRP) and D-dimer were more elevated in the IgM-/IgG- and IgM+ subgroups (P <0.0001 and p-value of 0.0452, respectively). The IgM+ group had the worst prognosis, with high mortality despite receiving remdesivir and dexamethasone.

Conclusion

Our findings suggest that the use of serology in patients hospitalized with COVID-19 could predict prognosis; this will need to be validated in a larger prospective study.

## Introduction

An estimated 2.74 million deaths were reported worldwide a year after the coronavirus disease 2019 (COVID-19) pandemic emerged [1]; multiple risk factors for mortality have been identified, but it is still unclear why some patients have a worse prognosis compared to others. It is well-established that patients with several comorbidities and those who suffer from a “cytokine storm” have worse disease progression and outcome [2]. The current Infectious Diseases Society of America (IDSA) guidelines recommend the use of certain drugs based on disease severity determined by oxygen saturation cutoffs, oxygen requirements, time since onset of symptoms, and imaging features [3]. Furthermore, the IDSA guidelines recommend the use of severe acute respiratory syndrome coronavirus 2 (SARS-CoV-2) nucleic acid amplification tests (NAAT) for the clinical diagnosis of COVID-19 and according to them, it is not recommended to use serologic testing for diagnosis or prognosis during the first two weeks after symptoms onset [4-5]. If serology is required for clinical or epidemiological purposes, it is recommended by the IDSA to test for immunoglobulin G (IgG) antibodies three to four weeks after symptom onset. Finally, regarding IgM antibodies, IDSA has no recommendations for or against using IgM antibodies to detect early infection [5].

Many established viral infections are diagnosed and managed according to their serologies and their time since the onset of symptoms. For example, hepatitis B virus infection is diagnosed and managed based on whether patients have positive hepatitis B core IgM or not. Moreover, studies of influenza virus suggest that early treatment with oseltamivir is more efficacious [6-9]. Regarding COVID-19 and remdesivir use, for example, the IDSA guidelines have not been using serologies or time since the onset of symptoms as determinants for eligibility. It is currently recommended to use remdesivir in patients with severe COVID-19 that is defined as oxygen saturation (SpO2) < 94% on room air, or in those requiring supplemental oxygen, mechanical ventilation, or extracorporeal mechanical oxygenation (ECMO) [10-12].

It is currently well-established that SARS-CoV-2 clinical manifestations vary with age and comorbid conditions; it covers a wide range of symptoms, spanning from asymptomatic to severe organ damage and death. The first stage of COVID-19 disease is the attachment of the virus to the angiotensin-converting enzyme (ACE) 2 receptors mainly in the respiratory system, followed by an immune response that often results in clearing of the infection; in some instances, this immune reaction leads to a “cytokine storm” with persistent fever, worsening shortness of breath, and multiorgan involvement. It is also well accepted that a hypercoagulable state can develop later during the disease. Although inflammatory markers like C-reactive protein (CRP) elevation was found to correlate with the cytokine phase, and, probably, the D-dimer level would correlate with a hypercoagulable state, it could be helpful for clinicians to recognize, with as much accuracy as possible, the stage of the disease the patient is currently in when managing such complicated illness, in order to optimize their treatment.

## Materials and methods

Study design

This was a single-center, retrospective cohort study conducted in a community hospital in Newark, New Jersey, United States. It was designed to correlate the stage of disease with patient prognosis. Hospitalized patients were eligible for the study if they had a clinically suspected or laboratory-confirmed SARS-CoV-2 infection with rapid antigen test and reverse transcription-polymerase chain reaction (RT-PCR) along with COVID-19 serologies. The Cellex qSARS-CoV2 IgG/IgM rapid antigen test (Cellex Inc., Research Triangle, North Carolina) was used as a qualitative detection of IgM and IgG antibodies against SARS-CoV-2 in blood, which has been granted Emergency Use Authorization [13-14]. The serology obtained allowed us to divide the stages of COVID-19 into acute (IgM/IgG negative), subacute (IgM positive), and late disease (IgM negative/ IgG positive). Along with the serology, the cause of death was obtained as well.

Inclusion/exclusion criteria

Patients that were included in the study were age ≥ 18 years old and hospitalized from October 1, 2020, to February 28, 2021, with suspected or laboratory-confirmed SARS-CoV-2 infection with a Cellex qSARS-CoV-2 IgG/IgM rapid test. Patients who did not receive COVID-19 serologies and had no clear clinical outcomes were excluded from the study.

Data collection and outcome

We collected patient data using electronic medical records. After screening the qualifying patients, we went through individual demographics, including age, sex, race/ethnicity, and comorbidities, including body mass index (BMI), diabetes mellitus, hypertension, chronic kidney disease (stage 3b-5/glomerular filtration rate <44). Further data were collected in regard to how many days of symptoms prior to admission, serology outcomes along with inflammatory markers, including lactate dehydrogenase (LDH), CRP, ferritin, and D-dimer, on the appropriate patients and was analyzed.

The primary outcome was all-cause mortality while being hospitalized in the various stages of the disease. Secondary outcomes included correlation with the inflammatory markers and the treatment correlation with the various stages of disease and review of the cause of death.

Statistical analysis

The patients’ demographics, clinical characteristics, serology, and biochemical data were retrieved and analyzed. Descriptive data are represented by mean ± standard deviation, percentage, numbers, and interquartile range. The Mann-Whitney test and T-test were adopted for non-normal and normal distribution of continuous variables, respectively. The analysis of variance (ANOVA) test was performed when comparing the continuous variables across multiple groups. As for categorical variables, the chi-square (χ2) or Fisher exact test was used. Correlation analysis was also performed to assess the severity of different stages of the COVID-19 disease with clinical outcome (need of mechanical ventilation and survival) based on the serology. All data analyses were performed with statistical software GraphPad Prism version 9.0.2 (GraphPad Software, San Diego, CA). Statistical significance was achieved if the null hypothesis could be rejected at a p-value of <0.05 with a 95% confidence interval. 

Ethical issues and informed consent

The retrospective study protocol has been approved by the Ethical Review Board of Saint Michael’s Medical Center, New York Medical College. A waiver of HIPAA privacy authorization has been obtained through the ethical review board. All procedures of the present study were conducted in compliance with the ethical standards of our institution as well as the Helsinki declaration for research on human beings.

## Results

A total of 252 patients admitted to the hospital for suspected COVID-19 between October 1, 2020, and February 28, 2021, had SARS-CoV-2 serology available. Patients with no clear clinical outcomes and negative serologies with negative PCR and antigen were excluded. A total of 202 patients met the inclusion criteria and were selected for the study. Duplicates were also excluded.

Demographics and baseline characteristics

Out of the 202 patients, 57% of the patients were males and 43% were females. The greatest percentage of patients were Hispanic (45%), 22% were African American, 15% were Caucasian, and 18% were of unknown race. Ninety-three patients had a body mass index (BMI) above 30 kg/m^2^ 74 had a BMI between 25 and 29.9 kg/m^2^; 28 patients were between 18.5-24.9 kg/m^2^; and only seven patients had a BMI below 18.5 kg/m^2^. Out of 202 patients, 91 were diagnosed with diabetes mellitus, 123 patients had hypertension, and only 24 patients had chronic kidney disease.

**Figure 1:**
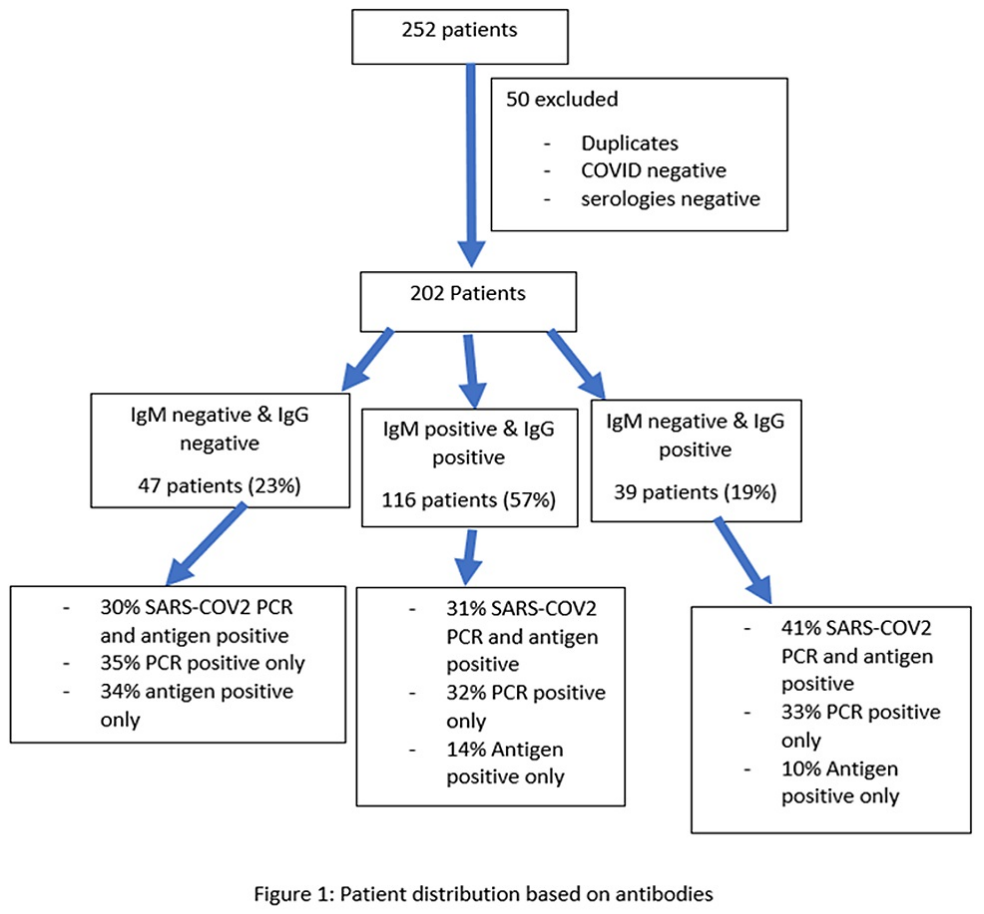
Patient distribution based on antibodies

The Cellex qSARS-COV2 IgG/IgM rapid test was used for serological testing. The patients were divided into three categories based on the serological results. Nineteen percent (19%) of the patients were IgM+ and IgG -, 57% of the patients were IgM+, whereas 23% of the patients were IgM- and IgG -. Out of the 47 patients who were IgM-/ IgG-, 30% were both SARS-CoV-2 PCR and antigen-positive, 35% were only PCR-positive, and 34% were antigen-positive. The patients' distribution based on serology are summarized in Figure 1.

Forty percent (40%) of the IgG-/IgM- group were females with a mean age of (SD), 36% were African Americans followed closely by Hispanics at 34%, 19 patients had a BMI above 30 and only four patients had a BMI below 18.5. In the IgG-/IgM- group, 19 patients were diagnosed cases of diabetes mellitus, 37 patients had hypertension, and 10 patients had chronic kidney disease. The IgM-positive subgroup had 116 patients, out of which 31% were both PCR and antigen-positive, 41% were females, and only eight patients were above 80 years of age. Based on race stratification, 51% were Hispanic, 18% were African American, and 13% were Caucasian. Fifty-five (55) patients were above 30 BMI, 63 patients had hypertension, 49 had diabetes, and 11 had chronic kidney disease. Forty-one percent (41%) of the 39 patients included in the IgM-/IgG + subgroup had positive SARS-CoV-2 PCR and antigen. Fifty-one percent (51%) of these patients were females, 38% were Hispanic, 21% were Caucasian, and 18% were African American. Nineteen (19) patients had a BMI above 30, and there were 23 patients with diagnosed diabetes mellitus and hypertension. The detailed demographic and clinical characteristics are summarized in Table 1.

**Table 1:** Detailed baseline characteristics across three groups BMI: body mass index

	Total	IgM-/IgG- (n=47)	IgM+ (116)	IgM-/IgG+ (n=39)
Age	60.92 +/- 14.98	64.70 +/- 15.66	58.98 +/- 14.66	60.56 +/- 14.51
Gender				
Male	116 (57%)	28 (60%)	69 (59%)	19 (49%)
Female	86 (43%)	19 (40%)	47 (41%)	20 (51%)
Ethnicities				
Blacks	45 (22%)	17 (36%)	21 (18%)	7 (18%)
Latinx	90 (45%)	16 (34%)	59 (51%)	15 (38%)
Whites	30 (15%)	7 (15%)	15 (13%)	8 (21%)
Others	37 (18%)	7 (15%)	21 (18%)	9 (23%)
Comorbidities				
Diabetes Mellitus	91 (45%)	19 (40%)	49 (42%)	23 (59%)
Hypertension	123 (61%)	37 (79%)	63 (54%)	23 (59%)
Chronic Kidney Disease	24 (12%)	10 (21%)	11 (9%)	3 (8%)
BMI	30.74 +/- 7.65	29.90 +/- 8.87	31.23 +/- 7.25	30.29 +/- 7.34
Symptoms onset before presentation	7.64 +/- 10.24	4.07 +/- 2.71	7.97 +/- 8.59	6.72 +/- 6.07
Oxygen saturation on presentation	92.83 +/- 9.49	95.40 +/- 4.53	92.05 +/- 8.20	95.40 +/- 4.53

The mean duration of symptoms for the IgM-/IgG- subgroup was 4.068 with SD 2.706 (interquartile range (IQR) 2-6.75 days), whereas the mean duration of symptoms for the IgM+ subgroup was 7.974 (SD 8.594) with an IQR 4-9 days and the mean duration of symptoms for the IgM-/IgG+ subgroup was 6.718 (SD 6.070, IQR 2-8 days). The mean oxygen saturation in the IgM-/IgG- subgroup was 95.40 with SD ± 4.53 (IQR: 93% -99%). The mean oxygen saturation for the IgM+ subgroup was 92.05 SD± 8.20 (IQR: 91% -96%). In the IgM-/IgG+ subgroup, the mean was 94.41 (SD 3.29, IQR: 92% -96%) (Table 1).

Inflammatory markers and stage of disease

**Figure 2:**
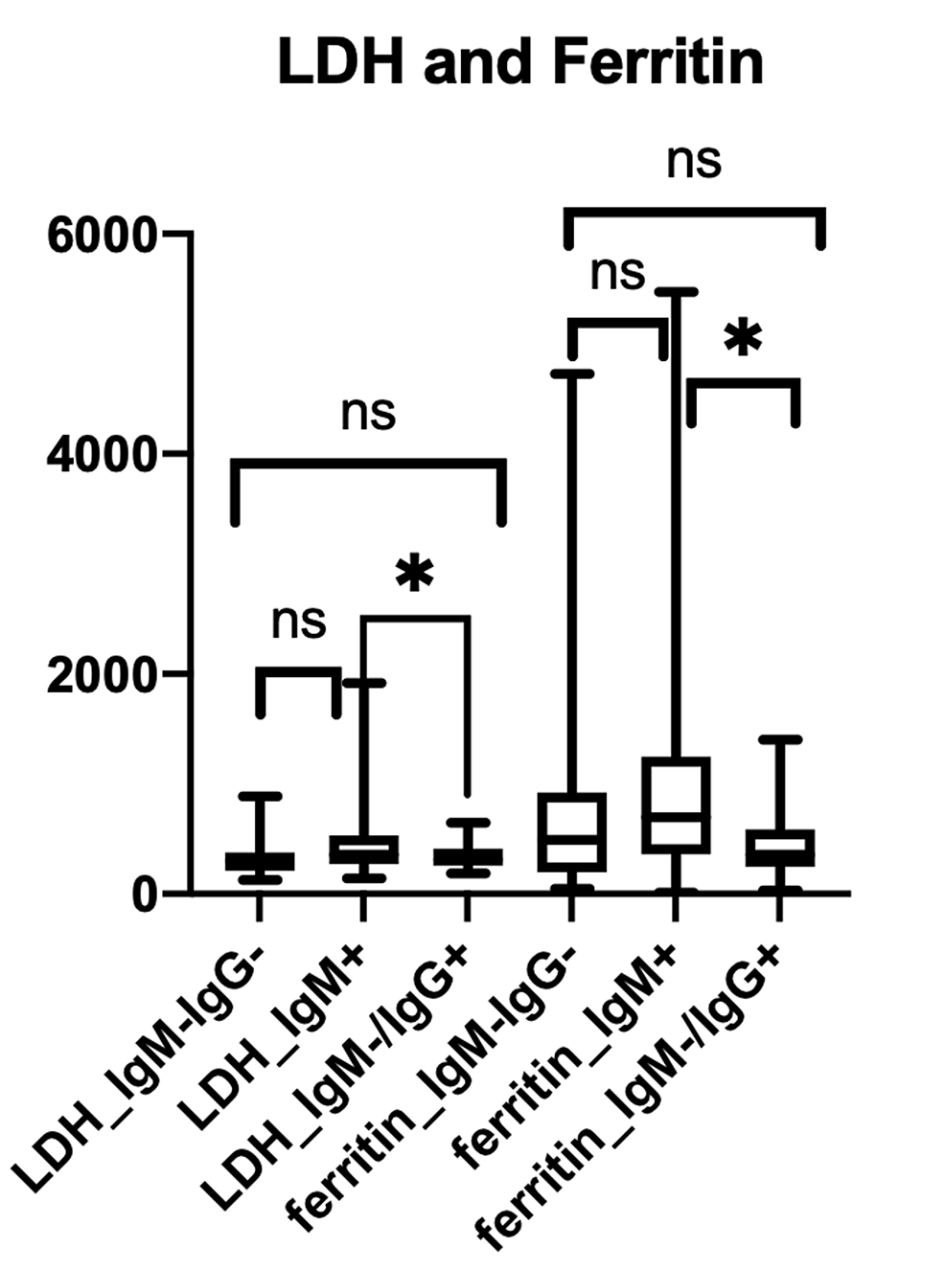
Inflammatory markers, including LDH and ferritin, across the three subgroups based on serological testing (IgG-/IgM-, IgM+, IgM-/IgG+) LDH: lactate dehydrogenase; Ig: immunoglobulin

The results of inflammatory markers, including LDH, ferritin, CRP, and D-dimer were compared across the three subgroups based on the serological testing (IgG-/IgM-, IgM+, IgG+/IgM-) using the ANOVA test. When comparing the LDH across the three groups, there was a statistically significant difference between the IgM+ and IgM-/IgG+ subgroups with a p-value of 0.0061. The comparison of ferritin across the three subgroups showed similar results with a statistically significant difference between the IgM+ and IgM-/IgG+ subgroups (p-value 0.0013) (Figure 2).

**Figure 3:**
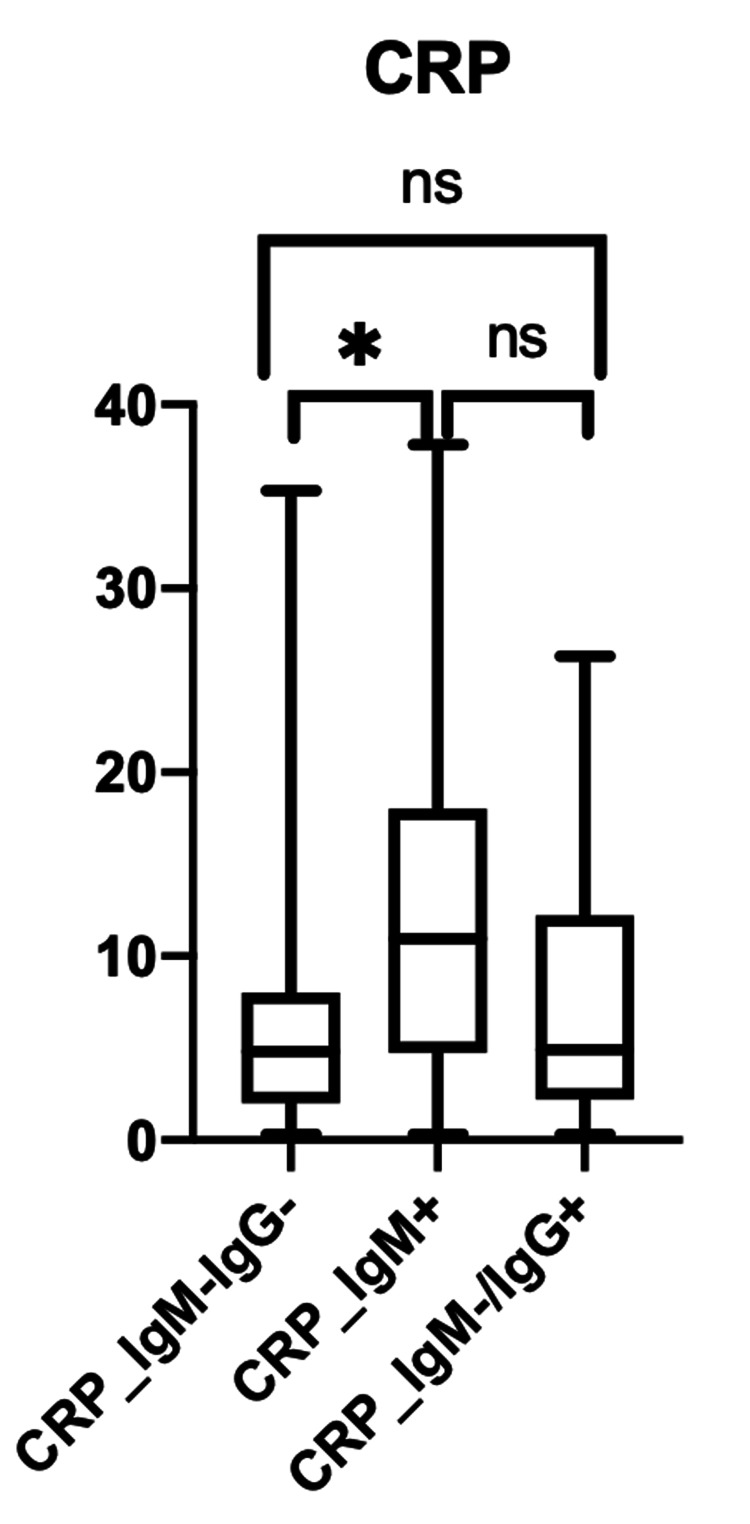
Inflammatory markers, including CRP, across the three subgroups based on serological testing (IgG-/IgM-, IgM+, IgM-/IgG+) CRP: C-reactive protein; Ig: immunoglobulin

The ANOVA for CRP showed a statistically significant difference between the IgM-/IgG- and IgM+ subgroups (P<0.0001) (Figure 3).

**Figure 4:**
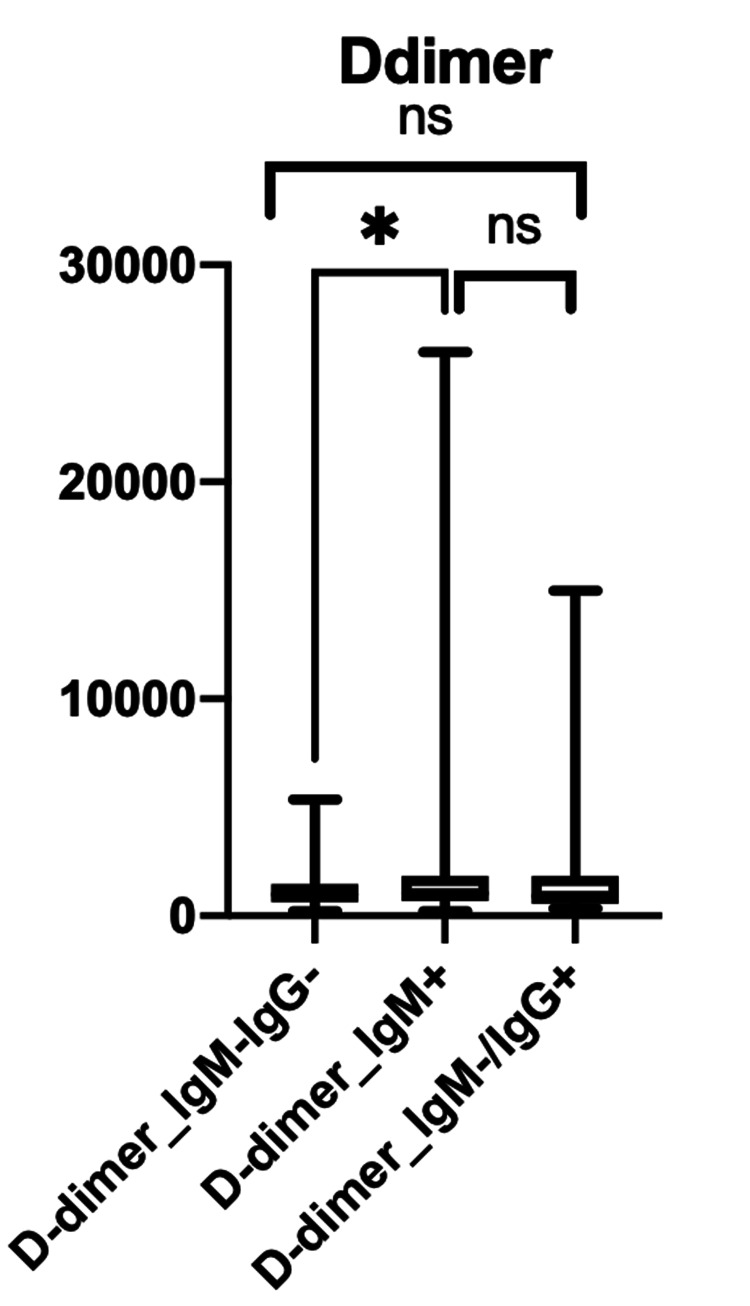
Inflammatory markers, including D-dimer, across the three subgroups based on serological testing (IgG-/IgM-, IgM+, IgM-/IgG+) Ig: immunoglobulin

The comparison between D-dimer showed a statistically significant difference between the IgM-/IgG- and IgM+ subgroups with a p-value of 0.0452 (Figure 4).

Treatment and clinical course

The most common medications used in these patients hospitalized for SARS-CoV-2 were remdesivir and dexamethasone. Analysis was done only in patients who received at least three days of a course of remdesivir. Sixty-six percent (66%) of the patients were in the IgM+ subgroup; 25% of these patients required mechanical ventilation with a survival percentage of 74%. Sixty-two percent (62%) were in the IgM-/IgG+ subgroup; only 4% required mechanical ventilation with no death. Forty-seven percent (47%) were in the IgM-/IgG- subgroup; 18% required mechanical ventilation in this subgroup and 25% of these patients expired. Dexamethasone was used in 55% of the patients in the IgM-/IgG- subgroup, 75% in the IgM+ subgroup, and 67% in the IgM-/IgG+ subgroup. The need for mechanical ventilation was most observed in the IgM+ subgroup (25%) out of which 77% expired. The use of remdesivir and clinical outcomes are summarized in Table 2.

**Table 2:** The use of remdesivir and/or decadron with the clinical outcomes across three groups RDV: remdesivir; MV: mechanical ventilation

		IgM-/IgG- (n=47)		IgM+ (116)		IgM-/IgG+ (n=39)	
		MV	No MV	MV	No MV	MV	No MV
RDV Only	Survived	-	1 (100%)	-	1 (100%)	-	-
	Expired	-	0 (0%)	-	0 (0%)	-	-
Decadron Only	Survived	-	4 (80%)	0 (0%)	9 (100%)	-	2 (100%)
	Expired	-	1 (20%)	3 (100%)	0 (0%)	-	0 (0%)
RDV and Decadron	Survived	3 (75%)	17 (100%)	5 (26%)	55 (98%)	0 (0%)	22 (96%)
	Expired	1 (25%)	0 (0%)	14 (74%)	1 (25)	1 (100%)	1 (4%)
Neither	Survived	1 (50%)	18 (100%)	1 (33%)	25 (100%)	1 (100%)	12 (100%)
	Expired	1 (50%)	0 (0%)	2 (67%)	0 (0%)	0 (0%)	0 (0%)

Outcome analysis

After analysis of the clinical outcome of the 202 patients, 16% of these patients required mechanical ventilation, whereas 84% of the patients were not intubated. Out of the 16% of patients who were intubated, 33% survived and 67% expired, whereas 98% of patients who did not require mechanical ventilation survived. Out of the 202 patients, only 12% expired and 88% survived. In the IgM-/IgG- group, 13% of the patients required mechanical ventilation and 67% of these patients survived. Twenty-two percent (22%) of the patients in the IgM+ group required mechanical ventilation and 25% of the patient on mechanical ventilation expired. Seventeen percent (17%) of the patients in the IgM+ group expired irrespective of the ventilation status. Only 5% of the patients required mechanical ventilation in the IgM-/IgG+ group. Ninety-five percent (95%) of the patients survived in the IgM-/IgG+ subgroup.

**Figure 5:**
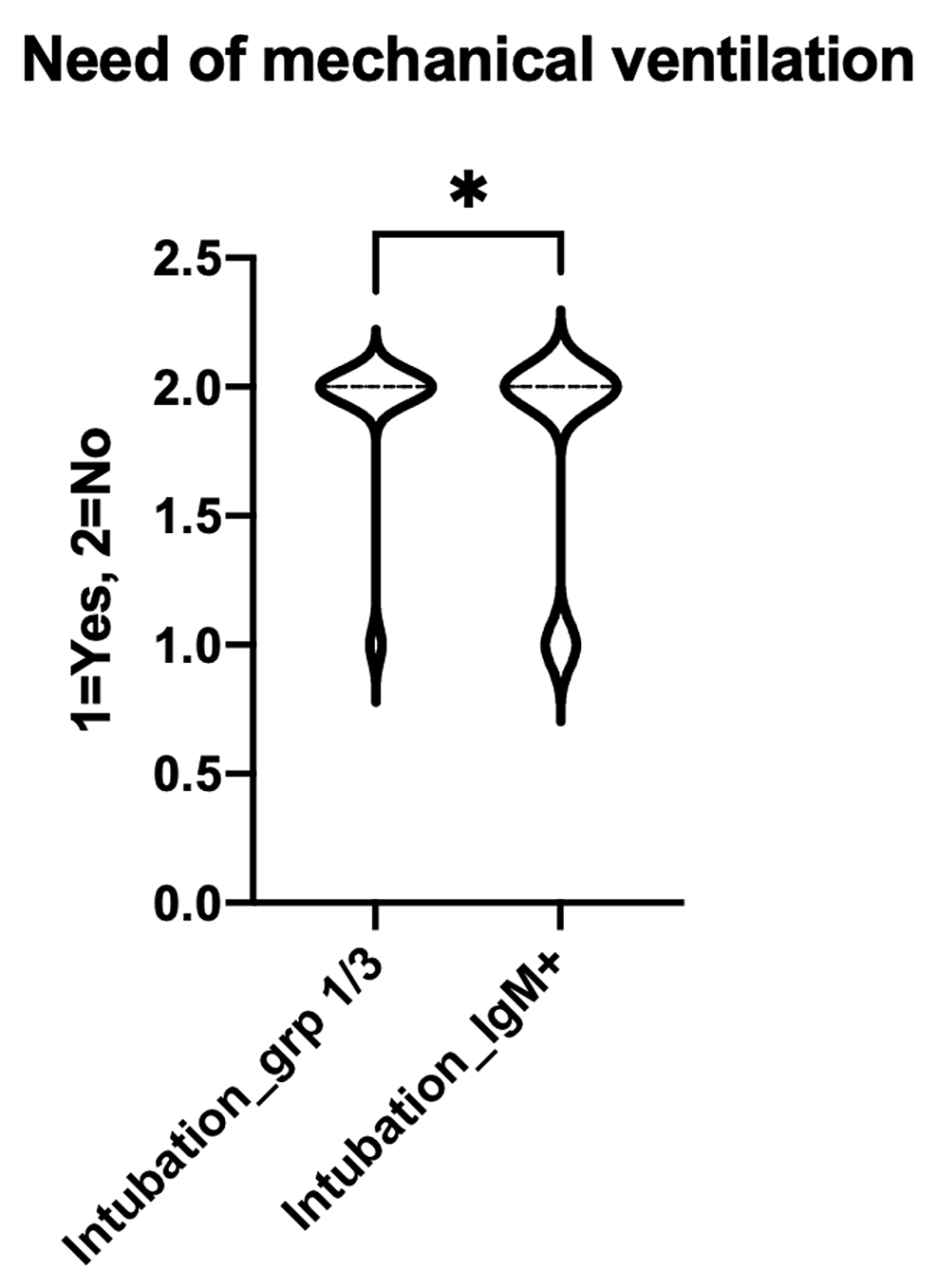
Comparing between the group 1&3 (IgM-/IgG- & IgM-/IgG+) with IgM+ on the need of mechanical ventilation

The need for mechanical ventilation was compared between the IgM-/IgG+ and IgM-/IgG- with the IgM+ subgroups, which was statistically significant (P-value 0.0198) (Figure 5).

**Figure 6:**
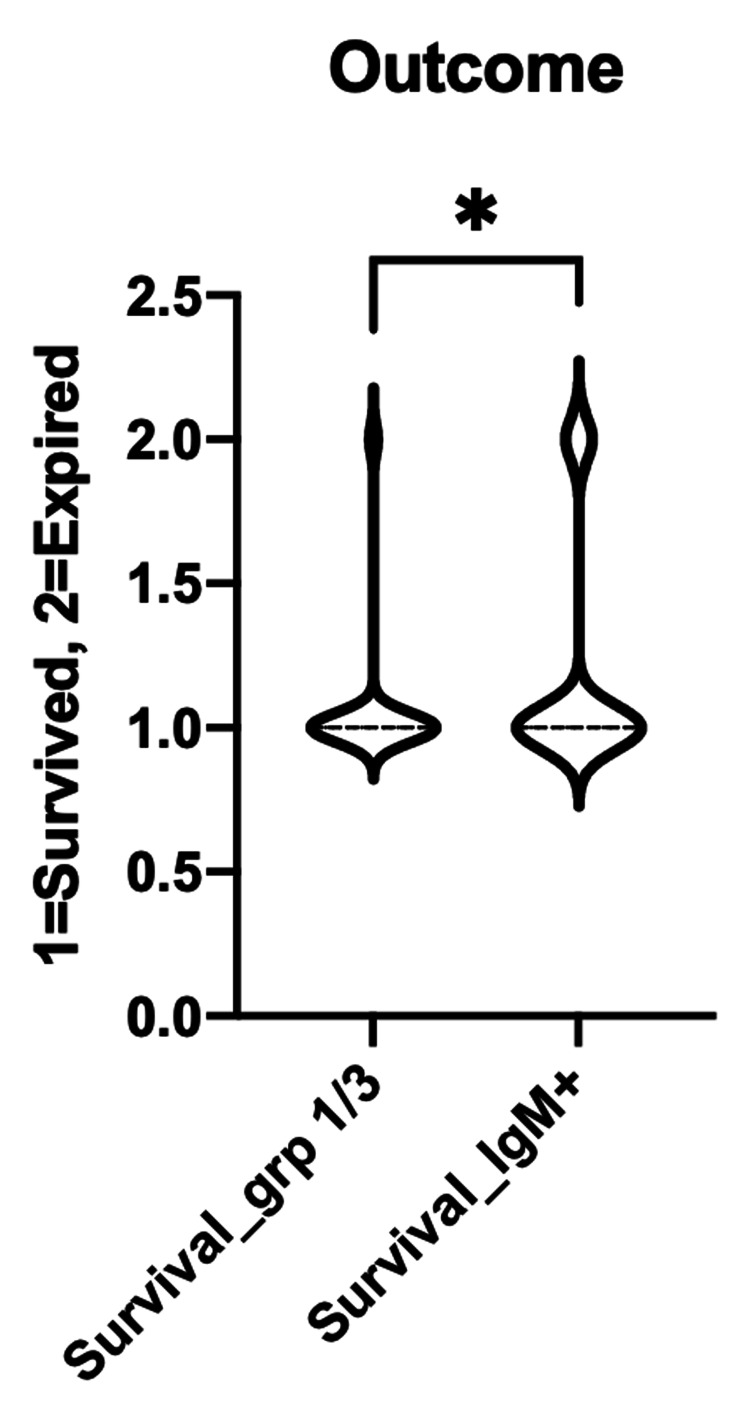
Comparing between groups 1 and 3 (IgM-/IgG- & IgM-/IgG+) with IgM+ on the clinical outcomes Ig: immunoglobulin

Similarly, the comparison of outcome between the IgM-/IgG+ and IgM-/IgG- with the IgM+ subgroup was also significant with a p-value of 0.0146 (Figure 6).

When associating inflammatory markers with the stage of disease, LDH and ferritin were statistically significant in subacute disease (IgM positive) and late disease (IgM negative/IgG positive) (p-value of 0.0061 and p-value of 0.0013, respectively) (Figure 2). The CRP and D-dimer showed a statistically significant difference between acute disease (IgM-/IgG-) and subacute disease (IgM+ subgroups) (P <0.0001 and p-value of 0.0452, respectively) (Figures 3-4).

Causes of death

The cause of death of the patients in the three groups was analyzed and identified. One of the patients with IgG and IgM negative had a history of chronic lymphocytic leukemia (CLL) and died due to the progression of viral pneumonia in the setting of an immunocompromised state. Another patient with IgG and IgM negative had a history of severe coronary artery disease (CAD) and died due to cardiac arrest. The third patient with IgG and IgM negative had uncontrolled HIV and died due to the progression of viral pneumonia. Of the two patients with IgM negative and IgG positive, one died of a cardiac arrest and the other patient refused mechanical ventilation and died of respiratory failure. In patients in the IgM positive subgroup, one died of respiratory failure with acute myocardial infarction (MI). Six patients died of respiratory failure with sepsis, seven patients died of respiratory failure with multiple organ failure, and one patient died of a cause unrelated to COVID-19 (Table 3).

**Table 3:** The causes of death across three groups. Ig: immunoglobulin

Cause of Death	IgM-/IgG- (n=47)	IgM+ (116)	IgM-/IgG+ (n=39)
Cardiac Arrest	1	1	1
Immunocompromised State	2		
Respiratory Failure With Sepsis		6	
Respiratory Failure With Multiorgan Failure		7	
Unrelated to COVID		1	

## Discussion

Our single-center retrospective study characterizes the outcomes of COVID-19 disease in relation to the stage of disease by serology. Since there are no current recommendations for COVID-19 serology use, this study can help to correlate mortality with early and late disease [5,15]. Antibody-based serologic tests measure the person’s humoral immune response, unlike NAAT, which detects viral RNA. Therefore, anti-SARS-CoV-2 antibodies typically become detectable more than two weeks after infection, which correlates with a viral replication phase of the disease [5,12,16-17].

IgM antibodies are used as a measure of recent infection since they are directed against microorganisms that are produced first after infection. IgG antibodies generally develop later after IgM and remain elevated for months to years after infection. Although IgM antibodies can be seen within the first two weeks of symptoms in some patients, SARS-CoV-2 infection is unique in that IgM and IgG more commonly rise together, more than two weeks after the onset of symptoms [18]. Liu et al. were able to observe that the IgM antibody peaked and was detected earlier on than the IgG antibody and that severe cases of COVID-19 had a more robust response in relation to IgG and IgM antibodies [13]. In our study, the IgM positive did show a clinical significance for increased mortality when compared with the other two groups.

Most therapeutic clinical trials for COVID-19 use oxygen saturation and time of symptoms onset for inclusion criteria as seen in the Adaptive COVID-19 Treatment Trial (ACTT-1) and SOLIDARITY trial. We believe that those two parameters may not always correlate with the different stages of the disease [19-20]. COVID-19 has been described as a viremic replication phase and an inflammatory phase. While in the early inflammatory phase, pulmonary involvement is evident, causing hypoxemia, which may require respiratory support [21-22]. During this phase, a cytokine storm occurs, which allows steroids to be beneficial in this stage and not earlier [23-25]. It has also been noted that the inflammatory markers, D-dimers, and other cytokines rise as well [26-27], like our study.

In our study, it showed that LDH and ferritin were statistically significant in the IgM+ and IgM-/IgG+ subgroups (p-value 0.0061 and p-value 0.0013, respectively). This demonstrated that LDH and ferritin are good inflammatory markers in the subacute and late stages of the disease process of COVID-19. However, CRP and D-dimer were statistically significant in the IgM-/IgG- and IgM+ subgroups (P <0.0001 and p-value of 0.0452, respectively), which shows that CRP and D-dimer are better inflammatory markers for acute and subacute disease. Overall, the elevation of all four inflammatory markers mentioned was seen in the IgM+ subgroup, which correlates with the present stage of the COVID-19 disease. Along with an increase in inflammatory markers, it was determined that IgM+ carried the worst prognosis, with high mortality despite receiving remdesivir and dexamethasone like in many instances [24,28-30]. This proves that clinical trials should be specifically addressing this group, and without serology, specifically IgM, it will be difficult to recognize those patients at high risk of a bad outcome.

The study has few limitations. First, this study was a single-center retrospective study. Second, the smaller sample size in individuals with serology. Due to the serology not being a recommendation, it was not used in the study center early on, therefore causing the sample size to be smaller, which may affect the power of the study. All future clinical trials should include multiple centers and be prospective studies to overcome these limitations. 

## Conclusions

Our study suggests that SARS-CoV-2 serology, including IgM and IgG, can be a helpful tool to guide the management of COVID-19. It also suggests that hospitalized patients with positive IgG and IgM are at higher risk for mortality than those with no antibody yet, or those who have lost their IgM despite the use of remdesivir and decadron. We couldn’t show the benefit of anti-cytokine therapy in this group because of the small sample size and the retrospective nature of our study. It is possible that such agents could decrease mortality in this subgroup of patients. We would advocate for a prospective randomized controlled trial of agents that could temper the inflammatory response beyond decadron for this subgroup of patients at the highest risk of death from COVID-19.
